# Model Gender Interacts With Expressed Emotion to Enhance Startle: Angry Male and Happy Female Faces Produce the Greatest Potentiation

**DOI:** 10.3389/fnhum.2020.576544

**Published:** 2020-11-09

**Authors:** Ole Åsli, Morten Øvervoll

**Affiliations:** Departmentof Psychology, UiT—The Arctic University of Norway, Tromsø, Norway

**Keywords:** startle, facial expressions, emotion, angry, happy

## Abstract

Several studies have implied gender differences in startle reaction to emotional facial expressions. However, few studies have been designed to investigate the difference between responding to emotional female vs. male faces, nor gender differences in responses. The present experiment investigated startle EMG responses to a startle probe while viewing pictures of neutral, happy, angry, fearful, and sad facial expressions presented by female and male models. Participants were divided into female and male groups. Results showed that emotional facial expressions interact with model gender to produce startle potentiation to a probe: greater responses were found while viewing angry expressions by male models, and while viewing happy faces by female models. There were no differences in responses between male and female participants. We argue that these findings underline theimportance of controlling for model gender in research using facial expressions as stimuli.

## Introduction

Visual stimuli are one of the most potent sources of information regarding our immediate environment. Emotional visual stimuli give us the information necessary for our well-being, and reactions to these stimuli have been investigated in numerous studies. In research, pictures containing emotional stimuli have been widely used to induce affect. The typical finding is that negative emotional pictures result in stronger reactions compared to neutral and positive images (Vrana et al., 1988; Cuthbert et al., 1996; Lang et al., 1998). Furthermore, emotional facial stimuli have often been included in the category of emotional scenes (Lang and Greenwald, 1988). However, the effects of emotional facial expressions are less consistent (Alpers et al., 2011; Paulus et al., 2014).

A relatively simple, but effective, tool for indexing emotional reactions is the startle eyeblink reflex. The startle reflex is a basic defensive reaction to sudden and intense stimuli (e.g., a loud noise). In humans, this involves, and is indexed by, the closure of the eyelids. It has been well documented that the amplitude of the startle eyeblink changes with the emotional stimuli viewed at the same time (Lang et al., 1990), and it seems to be caused by the priming of the startle circuitry by the amygdala (Davis, 1992). The typical emotional modulation is increased startle to (a startle probe while viewing) negative pictures and, to a lesser extent, inhibited responses to positive images (Vrana et al., 1988; Cuthbert et al., 1996; Lang et al., 1998; see also Bradley et al., 2006 on attentional modulation). However, research on startle while viewing emotional facial expressions has not revealed a clear pattern (Alpers et al., 2011; Paulus et al., 2014). Exposure of a startle probe while watching facial expression of anger seems to consistently potentiate the startle reaction (Balaban, 1995; Springer et al., 2007; Alpers et al., 2011; Åsli et al., 2017), but except from that, the question about which facial stimuli potentiates startle is still undetermined.

Several studies have found the mentioned effect of potentiated startle while watching angry facial expressions. Springer et al. (2007) found increased startle to angry faces compared to fearful, neutral, and happy faces. Similarly, Alpers et al. (2011) found increased startle to angry facial expressions compared to neutral. Also, Åsli et al. (2017) found potentiated startle to angry expressions compared to fearful and neutral expressions. Dunning et al. (2010) morphed pictures of faces and found potentiated startle to angry faces compared to neutral ones, but only for maximally angry expressions. Studying infants, Balaban (1995) found potentiated startle to angry faces. However, Waters et al. (2008) did not find modulated startles to angry facial expressions compared to neutral in 4- to 8-year-old children.

For the expression of fear, the results are less clear. Anokhin and Golosheykin (2010) found increased startle to fearful facial expressions compared to neutral and positive expressions. However, the fearful expressions were incorporated in the negative facial expressions category along with angry expressions. A tendency toward greater startle to fearful expressions compared to happy was reported by Springer et al. (2007). Also, Grillon and Charney (2011) found enhanced startle responses to fearful faces but only in a threat situation. For averted faces, increased startle has been found for fearful faces compared to angry and happy (Åsli et al., 2017). Potentiated startle to viewer directed angry faces and not to directed fearful faces could be explained by the fact that directed anger represent an unambiguous threat, whereas directed fear is more ambiguous and less immediately relevant (Springer et al., 2007).

For happy expressions, the results have been even more scarce. Alpers et al. (2011) reported a trend for potentiated startle to happy facial expressions compared to neutral. Both Alpers et al. (2011) and Åsli et al. (2017) found no difference between startle responses to happy and angry expressions. Interestingly, Alpers et al. (2011) used only female models, while Åsli et al. (2017) had both male and female models. Hess et al. (2007) found potentiated startle to female compared to male happy expressions. In a study, comparing facial expressions by in-group vs. outgroup members, Paulus et al. (2019) found increased startle to outgroup members showing smiles compared to in-group members showing the same emotion. In sum, it seems clear that other factors than emotional expressions, such as model gender, may play a decisive role in producing startle reactions.

A few studies have controlled for gender effects about startle reactions to emotional facial expressions. However, the ones which have included gender as a factor has generally found some effects. Hess et al. (2007) assessed startle eyeblink to happy, neutral, and angry expressions by men and women. They reported potentiated startle to angry male expressions, compared to neutral and happy, but no difference to female expressions. Similarly, Paulus et al. (2014) compared anger, fearful and neutral expressions by male and female models, and found startle potentiation to male angry, emotional faces compared to male fearful and neutral expressions. For female expressers, there were no differences in startle responding to the different expressions. It has been speculated that angry male expressions are more effective in signaling threat as they are socially dominant and therefore more legitimate (Hess et al., 2007).

Anokhin and Golosheykin (2010) examined the effect of participant gender. They found startle potentiation to negative facial expressions compared to neutral and happy expressions. However, when they analyzed the data separately by gender, the differences were only significant for female viewers. The results indicate that females may be more sensitive to emotional facial expressions compared to men and underlines the importance of analyzing the effect of participant gender. Based on the literature presented above, it seems vital to factor in both model and participant gender, to ensure that these factors do not cancel out the effect of the displayed emotion.

In the present study, we investigated the startle reflex during presentations of pictures displaying neutral, fearful, angry, happy, and sad facial expressions. Also, we assessed the effect of model gender and participant gender. Based on previous studies, we expected increased startle to anger, but possibly only male expressions of anger. For female expressions, we expected greater responses to happy faces compared to male faces with the same emotion. Besides, we controlled for any effects of participant gender by adding observer gender as a factor. In an exploratory part of the experiment, we included the facial expression of sadness. As we are not aware of any previous studies presenting this emotion, we had no specific hypothesis in that regard.

## Materials and Methods

### Participants

Forty-three individuals (21 men, 22 women, age range 19–35, mean age 23, 5 years) participated in the study. Subjective data were missing for eight participants. Data from two participants were excluded from startle analysis due to small responses. This left 35 participants for the subjective data analysis (18 men and 17 women), and 41 for the startle analysis (20 men and 21 women). All participants reported good health and did not report any consequential hearing problems, previous severe disease, or injury. The participants were instructed not to drink caffeinated beverages or use nicotine-containing substances for three hours before the experiment. They were also told that they could withdraw from the study at any time without giving any reason. Written informed consent was obtained from all participants, who were given two lottery tickets (equivalent to 50 NOK) for their participation or course credit for an introductory psychology class. The education level of the participants was not obtained.

### Apparatus and Stimuli

The experiment took place in an electrically and acoustically shielded chamber where the temperature was kept at about 20°C. A Bruel and Kjær 2235 Sound Level Precision Meter was used to measure the intensity of auditory stimuli. Programs for experimental control were written by the first author in Coulbourn Human Startle System HSW v. 7.500-00 and run on a Microsoft Windows XP based Dell PC that controlled presentation of experimental stimuli and data acquisition. The same software was used for scoring of startle responses.

Pictures of faces were randomly selected from the Radboud Faces Database (Langner et al., 2010) showing five emotions: neutral, fearful, angry, happy, and sad. Five females (model number: 1, 4, 57, 58, and 61) and five males (model number: 5, 20, 23, 38, and 71) expressers showed each facial expression, giving a total of five pictures of each facial expression-model gender combination. Pictures were presented for 5 s and in a random order (randomized for each participant). The intertrial interval (ITI) was between 17 and 23 s (mean 20 s). Color pictures were presented on a 17 in. monitor, subtended 17 and 11.3 degrees of visual angle vertically and horizontally.

Startle-eliciting noise had an intensity of 95 dB (SPL), instantaneous rise time, and a duration of 50 ms. The stimuli were delivered through Sennheiser HD 250 headphones. The startle-eliciting noise was presented between 4,000 and 5,000 ms after picture onset (random for every trial). The startle-eliciting stimulus was presented at every trial (once per picture). Before the presentation of any pictures, five trials of startle-eliciting noise was presented to minimize the effect of habituation in picture trials.

Startle eyeblink electromyographic (EMG) responses were recorded from the right orbicularis oculi (for practical reasons) with two sintered-pellet silver chloride AgCl miniature electrodes (4 mm diameter) filled with Microlyte electrolyte gel (Coulbourn Instruments). Inter-electrode distance was 1.5–2 cm. The ground electrode was placed centrally on the forehead. A factor of 50,000 amplified the EMG signal and filtered (passing 13–1,000 Hz) by a Coulbourn V75-04 bioamplifier. The signal was rectified and integrated with a Coulbourn V76-24 contour-following integrator with a 10-ms time constant, and the output was sent to the PC *via* a LabLinc V interface. A 12 bits A/D board was used. Sampling on each trial began 100 ms before the onset of the startle stimulus and continued for 200 ms after the onset of the stimulus. The sampling rate was 1,000 Hz.

For the subjective data, participants used the mouse pointer to indicate the level of valence, arousal, and domination elicited by each picture using a visual analog scale (VAS) on a computer screen. Each participant viewed each picture for as long as they wanted before rating. The instruction read (for valence): “Please mark on the line below how positive or negative you feel after seeing the face;” for arousal, the endpoints were labeled “relaxed,” and “agitated,” and for domination, the endpoints were “submissive” and “dominant.” The response range for both was from 0–100 mm. The program for the VAS was written in, and controlled by, MATLAB version 8.3 with Psychophysics Toolbox (Brainard, 1997).

### Procedure

After arrival at the laboratory, the participants sat down in a desk chair, read and signed the Informed Consent Form. After that, the participants were lead into the experimental chamber and seated in a reclining chair. The subjects were informed of the general purpose of the study and about the stimuli and procedure. They were also told that they could withdraw from the study without giving any reason at any time. The skin below the participants’ right eye was cleaned with a swab containing alcohol and pumice, and the electrodes for measurement of the startle blink electromyography (EMG) were attached. The headphones were attached, and the experimental procedure was initiated as described in the “Apparatus and Stimuli” section. The door to the experimental chamber was closed during all stimulus presentation.

After the startle session, the participants rated the pictures (valence, arousal, and domination) on a computer in the room adjacent to the experimental chamber (more details in the “Apparatus and Stimuli” section). After the subjective test, the experiment was over, the participants received the lottery tickets and left.

### Response Scoring and Data Reduction

Startle blink reflexes were scored as the difference between the maximum amplitude of the EMG response in the window from 0–200 ms after noise onset, compared to the mean EMG level for the last 100 ms before the onset of the startle-eliciting noise on that trial. Startle amplitude values were T-transformed (Z-scores multiplied by 10 and add 50). The startle baseline on each trial was calculated as the mean EMG activity in the last 100 ms before the startle-eliciting stimulus. Values below 20 A/D-units were scored as a non-response. The average for each picture type excluded values of zeroes (nonresponse trials). Less than 7% of startle responses were scored as nonresponses. Following these criteria, two participants were excluded from startle analysis. One because of nonresponses on more than 50% of the trials and one because of nonresponses on four of five trials to one picture type.

### Design and Analysis

The design was a two model gender (male, female) by five emotion (neutral, fearful, angry, happy, sad) by two participant gender (men, women) mixed design, where the two first factors were within factors and the last was a between factor. Results were considered significant if *p* < 0.05. Significant main effects or interactions related to the hypothesis were followed-up by contrast analyses.

## Results

### Startle

**Figure 1:**
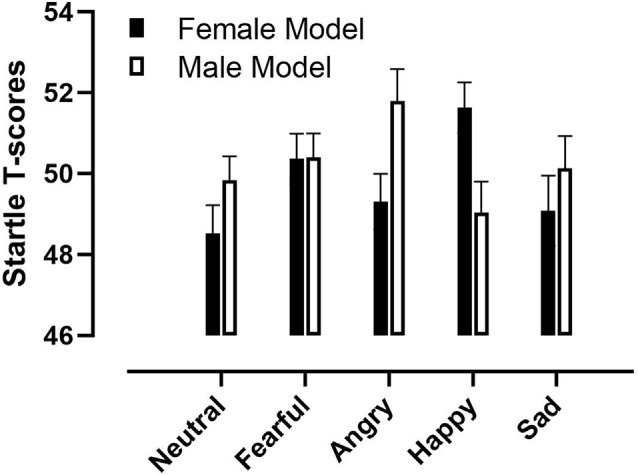
Startle response elicited during the viewing of pictures of different facial expressions showed by male and female models. Error bars represent +1 standard error of the mean.

There was a significant interaction of Model Gender by Emotion (*F*_(4,156)_ = 3.02, *p* = 0.02, $\eta_{\text{p}}^{2}$ = 0.07; Figure 1). The interaction of Participant Gender by Emotion was not significant (*F*_(4,156)_ = 1.14, *p* = 0.34, $\eta_{\text{p}}^{2}$ = 0.0004). Neither was any other main effect or interactions.

Following up the interaction, contrast analysis revealed the following differences: There was greater startle to male angry facial expressions compared to male happy faces (*F*_(1,39)_ = 5.51, *p* = 0.02, $\eta_{\text{p}}^{2}$ = 0.12). The difference between male angry faces and male neutral expressions was not significant (*F*_(1,39)_ = 3.64, *p* = 0.06, $\eta_{\text{p}}^{2}$ = 0.09).

Planned comparisons showed greater startle to female happy facial expressions compared to female neutral faces (*F*_(1,39)_ = 11.13, *p* = 0.002, $\eta_{\text{p}}^{2}$ = 0.22), female angry expressions (*F*_(1,39)_ = 6.16, *p* = 0.02, $\eta_{\text{p}}^{2}$ = 0.14), and female sad faces (*F*_(1,39)_ = 4.16, *p* = 0.05, $\eta_{\text{p}}^{2}$ = 0.10).

Regarding the difference between male and female expressions the following interesting differences was revealed: Greater startle to female happy expressions compared to male happy (*F*_(1,39)_ = 5.21, *p* = 0.03, $\eta_{\text{p}}^{2}$ = 0.12). Greater startle to male angry faces compared to female angry (*F*_(1,39)_ = 4.92, *p* = 0.03, $\eta_{\text{p}}^{2}$ = 0.11).

### Self-reported Ratings

#### Valence

**Figure 2:**
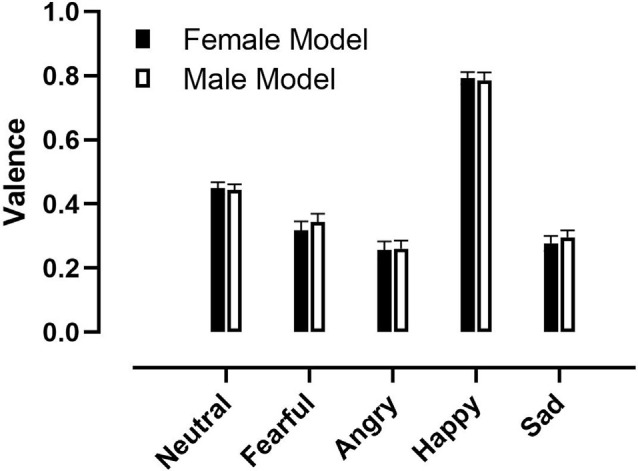
Valence ratings to pictures of different facial expressions showed by male and female models. Error bars represent +1 standard error of the mean.

There was a significant main effect of Emotion (*F*_(4,132)_ = 158.87, *p* < 0.001, $\eta_{\text{p}}^{2}$ = 0.82; Figure 2). Planned comparisons contrast analysis revealed that participants reported feeling more positive to happy faces, compared to neutral (*F*_(1,33)_ = 280.23, *p* < 0.001, $\eta_{\text{p}}^{2}$ = 0.89), fearful (*F*_(1,33)_ = 158.16, *p* < 0.001, $\eta_{\text{p}}^{2}$ = 0.83), angry (*F*_(1,33)_ = 224.68, *p* < 0.001, $\eta_{\text{p}}^{2}$ = 0.87) and sad faces (*F*_(1,33)_ = 214.56, *p* < 0.001, $\eta_{\text{p}}^{2}$ = 0.87). In addition, they reported feeling more positive to neutral faces than fearful (*F*_(1,33)_ = 26.43, *p* < 0.001, $\eta_{\text{p}}^{2}$ = 0.44), angry (*F*_(1,33)_ = 90.81, *p* < 0.001, $\eta_{\text{p}}^{2}$ = 0.73) and sad (*F*_(1,33)_ = 71.76, *p* < 0.001, $\eta_{\text{p}}^{2}$ = 0.69) faces. Participants also reported more positive valence to fearful faces compared to angry (*F*_(1,33)_ = 28.08, *p* < 0.001, $\eta_{\text{p}}^{2}$ = 0.46) and sad faces (*F*_(1,33)_ = 8.60, *p* < 0.001, $\eta_{\text{p}}^{2}$ = 0.21). Finally, they reported feeling more positive to sad faces than angry (*F*_(1,33)_ = 7.53, *p* = 0.009, $\eta_{\text{p}}^{2}$ = 0.19).

There were no other significant main effects or interactions.

#### Arousal

**Figure 3:**
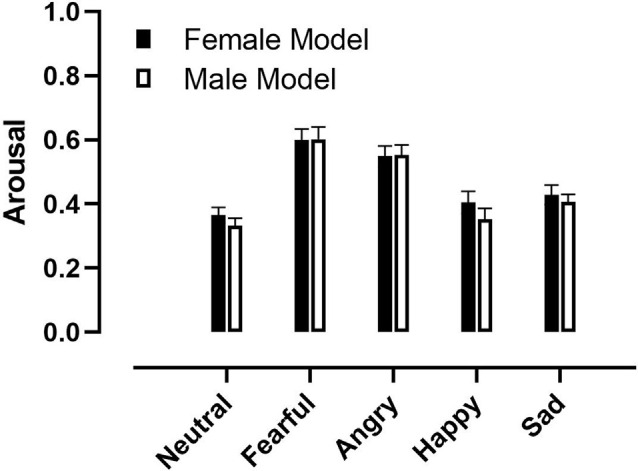
Arousal ratings to pictures of different facial expressions showed by male and female models. Error bars represent +1 standard error of the mean.

There was a significant main effect of Model Gender (*F*_(1,33)_ = 7.14, *p* = 0.01, $\eta_{\text{p}}^{2}$ = 0.17; Figure 3), as participants reported feeling more arousal to pictures of female models compared to pictures of male models.

In addition, there was a significant main effect of Emotion (*F*_(4,132)_ = 30.41, *p* < 0.001, $\eta_{\text{p}}^{2}$ = 0.46). Planned comparisons contrast analysis revealed that participants reported feeling more aroused to fearful faces, compared to neutral (*F*_(1,33)_ = 63.36, *p* < 0.001, $\eta_{\text{p}}^{2}$ = 0.66), angry (*F*_(1,33)_ = 22.35, *p* < 0.001, $\eta_{\text{p}}^{2}$ = 0.40), happy (*F*_(1,33)_ = 46.22, *p* < 0.001, $\eta_{\text{p}}^{2}$ = 0.58) and sad (*F*_(1,33)_ = 51.51, *p* < 0.001, $\eta_{\text{p}}^{2}$ = 0.61) faces. In addition, they reported feeling more aroused to angry faces compared to neutral (*F*_(1,33)_ = 55, 43, *p* < 0.001, $\eta_{\text{p}}^{2}$ = 0.63), happy (*F*_(1,33)_ = 24.01, *p* < 0.001, $\eta_{\text{p}}^{2}$ = 0.42) and sad (*F*_(1,33)_ = 39.73, *p* < 0.001, $\eta_{\text{p}}^{2}$ = 0.55) faces.

There was a significant interaction of Emotion by Participant Gender (*F*_(4,132)_ = 2.97, *p* = 0.02, $\eta_{\text{p}}^{2}$ = 0.04). Following up the interaction contrast analysis revealed no difference between Participant Gender on any of the facial expressions, however there was a trend toward larger arousal by females compared to males to sad faces (*F*_(1,33)_ = 3.23, *p* = 0.08, $\eta_{\text{p}}^{2}$ = 0.09).

In addition, there was a significant interaction of Model Gender by Emotion by Participant Gender (*F*_(4,132)_ = 2.45, *p* = 0.05, $\eta_{\text{p}}^{2}$ = 0.07). Contrast analysis revealed no difference between Participant Gender or Model Gender concerning any of the facial expressions.

There were no other significant main effects or interactions.

#### Dominance

There was a significant main effect of Participant Gender (*F*_(1,33)_ = 5.26, *p* = 0.03, $\eta_{\text{p}}^{2}$ = 0.14), as male participants reported feeling more dominant compared to female participants. There were no other significant main effects or interactions.

## Discussion

Two main findings emerged in the present study: Startle was potentiated to angry male faces and happy female faces. Angry male faces produced significantly increased startle compared to happy male faces, and angry female faces. Happy female faces significantly increased startle compared to female neutral, angry, and sad faces. Also, happy female faces produced greater startle compared to happy male faces. This is one of the first studies, to systematically investigate both participant gender and model gender concerning emotional expression in startle responding.

Increased reactions to angry facial expressions are the typical finding in research utilizing startle as the outcome variable (Balaban, 1995; Springer et al., 2007; Alpers et al., 2011; Åsli et al., 2017). However, some studies have found this modulation only with male models (Hess et al., 2007; Paulus et al., 2014), and Anokhin and Golosheykin (2010) found it only for female viewers. Regardless, potentiated startle to angry facial expressions stands out as the most reliable finding in research employing startle responding to measure reactions to emotional facial expressions. It is, however, worth noting that the difference in stimuli utilized in the different studies may account for the different results. As humans are experts at reading faces, small differences in stimuli material may account for pronounced differences in results.

Potentiated startle to angry male faces, but not to the same expressions shown by females, is an interesting finding. The same results were reported by Paulus et al. (2014). They argued that the reason for this was that an emotional face holds more social information than solely the emotional expression. More specifically, men are typically perceived as more dominant than women, and anger expressions showed by men should, therefore, more strongly activate the defensive motivational system. However, the data for dominance was collected based on a question about “how dominant the participant felt” while viewing the different pictures. As such, we cannot tell if the startle was potentiated to angry male faces because these pictures were perceived as more dominant. Besides, as happy female faces also potentiated startle, this explanation cannot be the only one for the present startle results.

Another possible explanation for the finding that only angry male faces potentiate startle is that angry male faces are perceived as angrier than angry female expressions. That is, maybe only angry male expressions are extreme enough to produce startle potentiation. If so, this would be in line with Dunning et al. (2010) who reported potentiated startle to morphed angry expressions but only with maximally angry faces. However, as there was no difference in valence ratings for female and male expressions, in the present study, this explanation is not likely.

Startle potentiation to happy faces has been less investigated (Hess et al., 2007; Springer et al., 2007; Anokhin and Golosheykin, 2010; Alpers et al., 2011; Duval et al., 2013; Åsli et al., 2017). Of these, only two studies have looked at the effect of happy faces displayed by female models *per se* (Hess et al., 2007; Alpers et al., 2011). Alpers et al. (2011) revealed, in a study using only female models, a trend for potentiated startle to happy facial expressions compared to neutral. Also, Hess et al. (2007) found potentiated startle to female compared to male happy expressions. Although, for female models, there were no differences between the different emotional expressions. In other words, previous research on startle responding to happy female faces shows varying results, however, the results from the present study seem to confirm a previously shown tendency of potentiated startle to happy female faces.

Facial stimuli also convey social meaning. Hence, it is important to remember that, for the participants, the models are both strangers, and showing fake smiles. Fake smiles in the regard that they are told to smile, and are, probably, not experiencing the joy that merits the facial expression. As such, the participants are reacting to pictures of unknown women who are directing fake smiles their way. However, this would be true for both female and male models, so strangers, showing fake smiles cannot be the whole explanation. Nevertheless, in a study investigating the perceived genuineness of pictures of emotional facial expressions, Dawel et al. (2017) found that expressions portraying happiness were generally perceived as genuine, or at least not fake. However, two of the pictures used in the present study in the happy female category was in the top 10 list of fake happy female stimuli (Dawel et al., 2017). Hence, possibly the present results with an enhanced startle to female happy expressions were driven by these fake expressions.

Another possibility is that there are some differences between male and female smiles in general. What are these differences? For one, females tend to smile more than males (Briton and Hall, 1995) and are found to be more facially expressive for positive emotions (Dimberg and Lundquist, 1990). This could lead to the perception that female smiles are less informative and therefore less sincere. As indicated by the early report of Bugental et al. (1971), who claimed that female smiles are perceived as falsely positive, and more recently by Krumhuber et al. (2007) reporting that smiles shown by females models were judged as less authentic than those displayed by men. A finding was also reported by Hutson-Comeaux and Kelly (2002). Accordingly, it may be that female smiles, who also is from strangers, is a cause for vigilance. That is if it is unclear if the smile is a sign of happiness or something else, it may be best to be alert. In that regard, it is not unlikely that this vigilance could manifest as a potentiated startle response given the link between the amygdala and the startle response (Davis, 1992; Angrilli et al., 1996). However, vigilance is typically not considered a part of the defensive system underlying startle responding.

Research in other domains has also singled out reactions to angry male faces and happy female faces. In reaction time studies participants are faster and more accurate detecting angry expressions on male faces and at detecting happy expressions on female faces (e.g., Becker et al., 2007). However, even though these results are commonly explained by the notion that the male faces look angrier and the female faces look happier, other explanations may be worth pursuing. Future studies should look more closely at the special effects of angry male and happy female faces, keeping in mind that it seems to produce unique reactions in multiple domains. In general, more research is needed on the interactions between the gender of the observer and the observed in various emotion paradigms, as pointed out by Kret and De Gelder (2012).

The difference between male angry faces and male neutral expressions was not significant, although there was a tendency for greater responses to angry male faces compared to neutral (*p* = 0.06). This tendency is in line with the typical finding (Balaban, 1995; Springer et al., 2007; Alpers et al., 2011). However, there are a few possible reasons why this effect did not reach significance in the present study. First, neutral facial expressions (or resting faces) are often perceived to show some emotion, and more often than not, a negative emotion (Hester, 2019). Second, and not necessarily unrelated, the difference in stimulus material may play a critical role here, as there seem to be individual differences in what emotion your neutral face emanate (Hester, 2019).

This discrepancy concerning model gender and emotional expressions underlines the importance of having expressers gender as a factor. Having both men and women as models, and treat them as identical stimuli, may severely distort the results in research on startle responding to facial stimuli. This may also explain the different results from previous studies. For instance, using an equal number of male and female stimuli would probably cancel out some of the effects seen in the present study, and using unequal numbers would possibly skew the data in the direction of the most typical findings of the overrepresented gender. Model gender should always be controlled for in future studies in this field: A conclusion that was also drawn by Duval et al. (2013).

As mentioned, the different results from the present study compared to earlier studies may be caused by the different pictures utilized. In the present study, pictures were chosen from the Radboud Faces Database (Langner et al., 2010), while the other studies mentioned used other picture sets. The Radboud Faces Database was created using the Directed Facial Action Task, in which the models are instructed to pose particular facial muscle configurations (Dawel et al., 2017). Facial expressions generated using this method are often perceived as fake, and Dawel et al. (2017) found that the categories of fear, anger, and sadness were perceived as fake overall. This points to the need for more research using genuine facial expression stimuli.

One other difference between earlier studies and the present is that the present study had more power, as it was designed to analyze the male and female groups separately. However, as there was no difference in startle reactions between male and female participants, the analysis profited from a large number of participants in the other analysis. Of course, this is also a strong point of the present study, as a larger sample increases the validity of the data.

Potentiated startle to smiling female faces compared to male happy, and angry male faces compared to female angry, stands in contrast to the subjective data. In the subjective data, there were no differences between ratings of valence, arousal, or dominance to these pictures. The reason behind this is not obvious, but it may that subjective data and startle data reflects different levels of processing (Sander et al., 2005). Previous research has shown that the startle response is modulated by negative stimuli even if participants are unable to consciously perceive their valence (Reagh and Knight, 2013). Paulus et al. (2014) reported similar results in that they found potentiated startle to angry male faces, but at the same time, these were not rated as more negative, arousing, or dominant than angry female faces. As suggested by Paulus et al. (2014), it may be that the startle response is sensitive and reflects processes unaware to the participants.

We did not find any effect of participant gender in the present study, in contrast to Anokhin and Golosheykin (2010). There were some differences between the two studies. First and foremost, Anokhin and Golosheykin (2010) used picture stimuli from Ekman’s and Friesen’s Pictures of Facial effect set (Ekman and Friesen, 1976), while the present study used the Radboud Faces Database (Langner et al., 2010). Also, in Anokhin and Golosheykin (2010) the startle probe was administered on two of three picture trials, whereas in the present study probes were presented on every trial. This could be a weakness to the present study; however, the predictability caused by this procedure would be the same for every trial and every picture type. Hence, it is unclear why it should affect the results.

Another limitation of the present study was the use of only startle eyeblink as a psychophysiological measure. Including other measures could have proven valuable in terms of explaining the results. Asking the participants to rate the genuineness of the stimuli would also have been beneficial. In future research using pictures of emotional facial expressions, this is recommended, as there may very well be a difference in how we react to genuine and fake expressions.

As an exploratory part of the study, we included the facial expression of sadness. Sadness is a negative emotion that is different from fear and anger in that it is much less arousing. It seems that there are stronger responses to male sad faces, compared to females, but this difference is not significant. However, it may be an idea to investigate this further in future studies with other stimuli materials.

In conclusion, the present study revealed that startle potentiation to emotional facial expressions depends on the gender of the model, while there was no effect of participant gender. Moreover, angry male faces potentiated startle responses, while angry female faces did not. For happy faces, the situation was reversed, as female happy faces potentiated startle, while happy male faces did not. These results underline the importance of controlling for model gender in research utilizing facial expressions of emotions as stimuli.

## Data Availability Statement

The raw data supporting the conclusions of this article will be made available by the authors, without undue reservation.

## Ethics Statement

Ethical review and approval was not required for the study on human participants in accordance with the local legislation and institutional requirements. The patients/participants provided their written informed consent to participate in this study.

## Author Contributions

OÅ developed the theoretical idea. OÅ and MØ planned the experimental protocol. MØ programmed the basic data collection procedure, while OÅ incorporated this into the final experimental setup. Both authors contributed to data analysis, and discussed the results. OÅ wrote the manuscript with support from MØ. All authors contributed to the article and approved the submitted version.

## Conflict of Interest

The authors declare that the research was conducted in the absence of any commercial or financial relationships that could be construed as a potential conflict of interest.
